# Examining Racial Disparities in Colorectal Cancer Screening and the Role of Online Medical Record Use: Findings From a Cross-Sectional Study of a National Survey

**DOI:** 10.2196/53229

**Published:** 2024-12-04

**Authors:** Aldenise P Ewing, Fode Tounkara, Daniel Marshall, Abhishek V Henry, Mahmoud Abdel-Rasoul, Skylar McElwain, Justice Clark, Jennifer L Hefner, Portia J Zaire, Timiya S Nolan, Willi L Tarver, Chyke A Doubeni

**Affiliations:** 1 College of Public Health, The Ohio State University, 1841 Neil Ave, 249 Cunz Hall, Columbus, OH, 43210, United States, 1 6146880748; 2 College of Medicine, The Ohio State University, Columbus, OH, United States; 3 Ohio Wesleyan University, Delaware, OH, United States; 4 College of Nursing, The Ohio State University, Columbus, OH, United States; 5 The University of Alabama at Birmingham, Birmingham, AL, United States

**Keywords:** colorectal cancer, cancer screening, early detection, Health Information National Trends Survey, cancer disparities, online medical records, secondary data analysis

## Abstract

**Background:**

Colorectal cancer (CRC) is the second leading cause of cancer-related deaths in the United States. Early detection via routine CRC screening can significantly lower risks for CRC-specific morbidity and mortality. Public health initiatives between 2000 and 2015 nearly doubled CRC screening rates for some US adults. However, screening rates remain lowest for adults aged 45‐49 years (20%), patients of safety net health care facilities (42%), adults without insurance (44%), and other subgroups compared with national averages (72%). Given the evolving landscape of digital health care and trends in web-based health information–seeking behaviors, leveraging online medical record (OMR) systems may be an underutilized resource to promote CRC screening utilization. Recognizing trends in OMR usage and patient demographics may enhance digital inclusion—a key social determinant of health—and support equitable web-based interventions aimed at boosting CRC screening across diverse populations.

**Objective:**

This study examined the association of accessing an OMR with CRC screening utilization and corresponding sociodemographic characteristics of US adults.

**Methods:**

In 2023, we conducted a secondary data analysis using a pooled, weighted sample from Health Information National Trends Survey (HINTS) 5 cycles, 2, 3, and 4 (2018‐2020), a nationally representative survey assessing how US adults access and use health-related information. We analyzed the association between sociodemographic characteristics, medical conditions, OMR access, and CRC screening behaviors via logistic regression.

**Results:**

The sample included adults aged 45‐75 years (N=5143). The mean age was 59 (SD 8) years for those who reported CRC screening and 52 (SD 6) years for those never screened. Nearly 70% (4029/5143) of participants reported CRC screening and 52% (2707/5143) reported OMR access in the past year. Adjusted odds of CRC screening were higher among non-Hispanic African American or Black adults than among non-Hispanic White adults (odds ratio [OR] 1.76, 95% CI 1.22‐2.53), adults who accessed an OMR (OR 1.89, 95% CI 1.45‐2.46), older individuals (OR 1.18, 95% CI 1.16‐1.21), the insured (OR 3.69, 95% CI 2.34‐5.82), and those with a professional or graduate degree versus those with a high school diploma or less (OR 2.65, 95% CI 1.28‐5.47). Individuals aged 65‐75 years were significantly more likely (*P*<.001) to be screened (1687/1831, 91%) than those aged 45‐49 years (190/610, 29%).

**Conclusions:**

Promoting OMR access, especially among the most disadvantaged Americans, may assist in reaching national screening goals. Emphasis should be placed on the mutability of OMR use compared with most other statistically significant associations with CRC screening behaviors. OMR access provides an intervenable means of promoting CRC education and screening, especially among those facing structural barriers to cancer diagnoses and care. Future research should focus on tailored and accessible interventions that expand OMR access, particularly for younger populations.

## Introduction

### Colorectal Cancer Screening Disparities

Due to initiatives in public health aimed at encouraging colorectal cancer (CRC) screening among demographics with historically low rates, the screening rates for CRC have more than doubled from 2000 to 2015 for non-Hispanic Black, Hispanic, and non-Hispanic Asian adults aged 50-75 years in the United States [1]. According to reports, CRC screening rates are now comparable for non-Hispanic Black adults (75%) and non-Hispanic White adults (74%), but lower for Hispanic adults (64%) and non-Hispanic Asian adults (61%) in the United States [2]. Furthermore, lower than national average rates of CRC screening (72%) are still reported among adults younger than 65 years (ie, pre-Medicare eligibility) and those who report less educational attainment than a college degree, are uninsured, and have recently immigrated to the United States [3-6]. CRC screening promotion remains critical, as adherence to recommendations by the United States Preventive Services Task Force (USPSTF)—recently updated in 2021—could prevent deaths or effectively treat at least 35,000 CRC diagnoses over the lifetime of age-eligible adults [7].

### CRC Screening Modalities

The USPSTF recommends various CRC screening methods for individuals at average risk and beginning at age 45 years [7]. These include stool-based tests and direct visualization techniques at respective intervals. Stool-based tests, which are done at home without the need for bowel preparation or anesthesia, include the annual guaiac-based fecal occult blood test, the annual fecal immunochemical test (FIT), and the FIT-sDNA test, administered every 1-3 years. A positive result from any of these tests requires a follow-up colonoscopy.

Direct visualization tests, such as colonoscopy every 10 years, computed tomographic colonography every 5 years, flexible sigmoidoscopy every 5 years, and flexible sigmoidoscopy (every 10 years) with an annual FIT, involve more invasive procedures such as bowel preparation and anesthesia. Any positive result from these tests, other than a colonoscopy, also necessitates a follow-up colonoscopy.

### Evidence-Based Initiatives for CRC Screening Promotion

Primary reasons for underutilization of CRC screening are low patient awareness of the importance and need for screening, poor access to regular medical care and screening tests, lack of insurance, and lack of health care provider recommendation for the test [8-13]. While physician recommendation is one of the strongest predictors of screening uptake, health care providers encounter time and resource constraints that limit their ability to effectively educate patients and discuss screening recommendations during visits [9-12]. As such, there has been a proliferation of interventions to increase CRC screening. These interventions have been directed at several levels including the client (eg, patient education, tailored or nontailored print media or videos, etc), the provider (eg, provider incentives, provider assessment and feedback, etc) and health system or organization (eg, client reminders, patient navigation, etc), or any combination of levels [14]. However, there still exists disparities in utilization of repeat CRC screening, CRC screening among “newly” screen age-eligible adults between 45 and 49 years of age, and CRC screening completion among adults who have received an abnormal stool-based test result [15-17]. Online medical records (OMRs), either as an educational tool for patients or as a clinical tool that enhances patient-provider communication, may be an existing and underutilized resource for promoting CRC screening interventions and addressing remaining disparities in CRC screening utilization across the continuum.

### OMRs for CRC Screening Promotion

Patient education and awareness concerning the importance of CRC screening remains a constant need, especially within US community clinics that report CRC screening rates as low as 43% [18-20]. Targeted web-based cancer education interventions may leverage growing trends in web-based health information–seeking behaviors—more than 60% of US adults report seeking web-based health information [21-24]. Leveraging existing platforms, including OMRs with embedded patient portals, could alleviate barriers to health care access and communication shortcomings to improve CRC screening completion rates [25-29]. OMRs have been used to remind patients about screening, refer patients to specialists, schedule appointments, and empower patients to take charge of their own care [30]. Furthermore, the integration of electronic health records with patient access to OMRs has been associated with improved CRC screening and other preventative health screenings [31]. Therefore, this study aims to investigate the association between OMR access and CRC screening behaviors among age-eligible adults in the United States, with particular emphasis on understanding racial and ethnic disparities. The goal is to explore whether OMR access can serve as an effective tool in promoting CRC screening. In addition, the study seeks to identify potential OMR-based interventions that could address existing disparities and improve CRC screening rates across diverse and socially vulnerable populations. Identifying growing OMR usage patterns and patient profiles could promote digital inclusion—a social determinant of health—and equitable web-based cancer education–based interventions to increase CRC screening among diverse groups [32].

## Methods

### Study Design and Data Source

Data for this secondary data analysis study were obtained from the Health Information National Trends Survey (HINTS) 5 cycles 2, 3, and 4, conducted between 2018 and 2020 [33]. Full details about HINTS methodology can be seen on the HINTS website [34]. HINTS is a nationally representative survey conducted by the National Cancer Institute to assess health communication, health information–seeking behaviors, and health-related attitudes and behaviors in the United States. The survey is designed to provide cross-sectional data that can inform cancer-related communication and health promotion efforts at a population level.

### Study Population

This study population consisted of nonincarcerated, US adults aged 45‐75 years (N=5143) who participated in the HINTS survey during the specified cycles. This age range was chosen to focus on the national population of average-risk adults recommended to undergo screening for CRC by the USPSTF [7].

### Ethical Considerations

The HINTS 5 survey, conducted with the general population, underwent expedited review and received approval from the Westat institutional review board (IRB) on March 28, 2016 (project no. 6048.14). In addition, on April 25, 2016, the National Institutes of Health Office of Human Subjects Research determined that the survey did not involve human subjects research, providing an exemption (exempt no. 13204) [35]. This analysis used deidentified, publicly available data from the HINTS, which did not constitute human subjects research as defined by 45 CFR 46.102 and, therefore, did not require IRB review. The original consent and IRB approval cover secondary analysis without the need for additional consent. No compensation was provided for participation.

### Measures

#### Primary Outcome

The primary outcome was assessed using the following survey item: “Have you ever had a test to check for colon cancer?” Responses were dichotomized as yes or “ever screened” and no or “never screened.” Participants were categorized as “ever screened” if they reported undergoing any CRC screening test in the past.

#### Independent Variables

Sociodemographic characteristics included age, gender, education level, income, and insurance status. Age was treated as a continuous variable, while gender was categorized as male or female. Education level was categorized into groups such as high school or less, some college, and postgraduate degree. Income was categorized into income brackets (eg, <US $20,000, US $20,000-US $35,000, etc), and insurance status was dichotomized as insured or uninsured.

Race and ethnicity were self-reported and categorized as non-Hispanic White, non-Hispanic Black, Hispanic, non-Hispanic Asian, and other.

Medical conditions were self-reported and included diabetes, high blood pressure, heart conditions, lung disease, depression, or family history of cancer. Responses were dichotomized as yes or no.

Accessing an OMR was assessed by asking participants whether they had accessed their OMR at least once in the last 12 months. This variable was dichotomized as yes or no.

### Statistical Analysis

#### Survey Weights

Survey weights were essential in the analysis of the HINTS dataset to account for the complex survey design and adjust for potential biases. The survey weights provided by HINTS were derived using the jackknife replication method and adjusted for selection bias resulting from the complex sampling design, nonresponse bias due to differential participation rates (ie, lower responses from men compared with women), and poststratification to align the sample with the population distribution by key sociodemographic characteristics. A method similar to the quasi-randomization approach was used for (HINTS) 5 cycles 2, 3, and 4 to adjust for household-level nonresponse. Adjustments were made for sample stratum (ie, lower response among those in high concentrations of minority populations), census region, address, metropolitical status, and high Spanish linguistically isolated areas [36-38].

Survey weights were applied to account for the complex survey design and produce generalizable population estimates. Weighted analyses were conducted, considering the appropriate weight variable provided by HINTS, to ensure that the results accurately reflected the target population of adults aged 45‐75 years in the United States.

#### Analysis

All statistical analyses were conducted in R Statistical Software (v4.1.2; R Core Team 2021). Descriptive statistics were reported to summarize characteristics of the study population. Frequencies and percentages were calculated for categorical variables, while means and SDs were computed for continuous variables. These descriptive statistics provided an overview of the sample and the distribution of key variables.

Bivariate analyses were conducted to examine the associations between the primary outcome (CRC screening) and various independent variables. Chi-square tests were performed to assess associations between CRC screening and variables of interest, such as race and ethnicity, accessing an OMR, sociodemographic characteristics, and medical conditions.

Multivariable logistic regression analysis was used to assess the independent associations between the primary outcome and key independent variables, while controlling for potential confounders. Missing data were handled using listwise deletion, excluding participants with missing values on any of the variables included in the models. This approach was chosen because the proportion of missing data for the primary outcome of interest was small (<5%), and no patterns of missingness were identified that would suggest systematic bias. Adjustments were made for relevant covariates, such as age, gender, education, income, and insurance status. The adjusted odds ratios and their corresponding 95% CIs were calculated to estimate the strength and direction of the associations between the independent variables and CRC screening. The multivariable logistic regression analysis allowed for the identification of significant predictors of CRC screening, considering the potential influence of confounding factors.

## Results

### Study Population Characteristics

Among the weighted sample (N=257,211,194), approximately 70% (4029/5143) of the participants reported having undergone CRC screening (Table 1). Most participants were non-Hispanic White (3527/5143, 72%). The mean age for individuals who reported CRC screening was 59 (SD 8) years, while it was 52 (SD 6) years for those who had never been screened (Table 2). A little more than half of the participants (2707/5143, 52%) reported accessing their OMR at least once in the past year. Table 2 presents the results of the bivariate analyses, examining the associations between CRC screening, various sociodemographic characteristics, medical condition variables, and main predictors (race and OMR access).

**Table 1.** Characteristics of main outcomes.

Characteristic (N=257,211,194)^a^		n (%)
**Colorectal cancer screening**		
	Ever screened	4029 (70)
	Never screened	1114 (30)
	Total	5143
**Race**		
	NH^b^ White	3527 (72)
	Hispanic	582 (12)
	NH Black or African American	674 (9.3)
	NH Asian	191 (4.3)
	NH other^c^	169 (2.7)
	Total	5143
**Access online medical record**		
	None	2436 (48)
	At least 1 time	2707 (52)
	Total	5143

a Weighted counts based on pooled sample of 5143 adult participants, derived using weights.

b NH: non-Hispanic.

c Includes non-Hispanic (NH) American Indian or Alaska Native, NH Native Hawaiian or other Pacific Islander, and NH Multiple Races Mentioned.

**Table 2.** Participant characteristics by colorectal cancer screening comparisons.

		Ever screened^a^	Never screened^a^	*P* value
**Race, n (%)**				<.001
	NH^b^ White	2820 (72)	707 (28)	
	Hispanic	412 (71)	170 (29)	
	NH Black or African American	544 (74)	130 (26)	
	NH Asian	130 (60)	61 (40)	
	NH other^c^	123 (60)	46 (40)	
**Access online medical record, n (%)**				<.001
	None	1748 (63)	688 (37)	
	At least 1 time	2281 (75)	426 (25)	
Age (years), mean (SD)		59 (8)	52 (6)	<.001
**Age group (years), n (%)**				<.001
	45‐49	190 (29)	420 (71)	
	50‐64	2152 (76)	550 (24)	
	65‐75	1687 (91)	144 (9)	
**Sex, n (%)**				.79
	Male	1752 (69)	451 (31)	
	Female	2271 (70)	659 (30)	
**Education, n (%)**				<.001
	High school or less	104 (52)	59 (48)	
	Post–high school/some college	522 (65)	186 (35)	
	College graduate	1306 (70)	345 (30)	
	Postgraduate	2090 (74)	523 (26)	
**Insurance, n (%)**				<.001
	No	66 (28)	109 (72)	
	Yes	3936 (72)	996 (28)	
	Missing	27 (71)	9 (29)	
**Income, n (%)**				.11
	<US $20,000	420 (65)	167 (35)	
	US $20,000-US $35,000	411 (65)	122 (35)	
	US $35,000-US $50,000	473 (65)	142 (35)	
	US $50,000-US $75,000	728 (71)	178 (29)	
	≥US $75,000	1723 (72)	443 (28)	
**Diabetes, n (%)**				.39
	No	3013 (69)	903 (31)	
	Yes	972 (72)	205 (28)	
	Missing	44 (77)	6 (23)	
**High BP** ^**d**^ **, n (%)**				<.001
	No	1890 (66)	675 (34)	
	Yes	2090 (73)	434 (27)	
	Missing	49 (89)	5 (11)	
**Heart condition, n (%)**				.24
	No	3578 (69)	1041 (31)	
	Yes	413 (75)	68 (25)	
	Missing	38 (80)	5 (20)	
**Lung disease, n (%)**				.07
	No	3443 (69)	996 (31)	
	Yes	553 (76)	113 (24)	
	Missing	33 (79)	5 (21)	
**Depression, n (%)**				.19
	No	3043 (69)	862 (31)	
	Yes	942 (72)	247 (28)	
	Missing	44 (87)	5 (13)	
**Family history of cancer, n (%)**				.33
	No	478 (65)	163 (35)	
	Yes	2037 (69)	529 (31)	
	Missing	121 (62)	43 (38)	

a Weighted percentages based on pooled sample of 5143 adult participants, derived using weights. Significant differences in CRC screening were evaluated with Rao-Scott tests for weighted data.

b NH: non-Hispanic.

c Includes non-Hispanic (NH) American Indian or Alaska Native, NH Native Hawaiian or other Pacific Islander, and NH Multiple Races Mentioned.

d BP: blood pressure.

### CRC Screening by Participant Characteristics

Overall, higher proportions of non-Hispanic White participants (2820/3527, 72%) and non-Hispanic Black participants (544/674, 74%) reported CRC screening, while only 54% (412/582) of Hispanic participants reported CRC screening (*P*<.001) (Table 2). Age was significantly associated with CRC screening, with older individuals having higher rates of screening (*P*<.001). Fewer participants between the ages of 45‐49 years reported CRC screening (190/610, 29%) compared with older age groups between the ages of 50‐64 years (2152/2702, 76%) and 65‐75 years (1687/1831, 92%) (*P*<.001). Higher educational attainment was significantly associated with CRC screening, with 52% (104/201) of participants with a high school degree or less screened, compared with 74% (2090/2813) of participants with postgraduate degrees (*P*<.001). Being insured was associated with CRC screening, with 72% (3936/4932) of insured participants reporting CRC screening compared with 28% (66/235) of uninsured participants (*P*<.001). Among participants with high blood pressure, 73% (2090/2524) reported CRC screening, while 66% (1890/2565) of participants with no high blood pressure reported CRC screening (*P*<.001).

### Accessing an OMR and Participant Characteristics

Table 3 shows results of the bivariate analyses, examining associations between accessing an OMR and race and ethnicity, as well as other sociodemographic characteristics and medical conditions. More than half of Hispanic participants (338/582, 62%) and non-Hispanic Black participants (343/674, 56%) reported no access to their OMR in the last 12 months, compared with 45% (1587/3527) of non-Hispanic White participants (*P*<.001). The proportion of participants who accessed their OMR at least once in the last 12 months increased with higher educational attainment, with 62% (1580/2613) of participants with postgraduate degrees accessing their OMR compared with 25% (35/163) of participants with a high school degree or less (*P*<.001). Gender was significantly associated with accessing an OMR, with 54% (1617/2930) of women accessing their OMR compared with 49% (1084/2203) of men (*P*=.02). Insurance status was strongly associated with accessing an OMR, with 53% (2662/4932) of insured participants accessing their OMR compared with 20% (36/235) of uninsured participants (*P*<.001). Higher proportions of participants with higher income reported accessing an OMR (*P*<.001). Participants reporting diabetes (658/1177, 57%) and high blood pressure (1361/2524, 55%) reported significantly higher OMR access than participants with no specified medical conditions (*P*=.02 and *P*=.03, respectively).

**Table 3.** Participant characteristics by online medical record access status.

		At least 1 time^a^	None^a^	*P* value
**Race, n (%)**				<.001
	NH^b^ White	1940 (55)	1587 (45)	
	Hispanic	244 (38)	338 (62)	
	NH Black or African American	331 (44)	343 (56)	
	NH Asian	102 (52)	89 (48)	
	NH other^c^	90 (52)	79 (48)	
Age (years), mean (SD)		57 (8)	57 (8)	.75
**Age group (years), n (%)**				.87
	45‐49	306 (51)	304 (49)	
	50‐64	1416 (51)	1286 (49)	
	65‐75	985 (53)	846 (47)	
**Sex, n (%)**				.02
	Male	1084 (49)	1119 (51)	
	Female	1617 (54)	1313 (46)	
**Education, n (%)**				<.001
	High school or less	35 (25)	128 (75)	
	Post–high school/some college	283 (39)	425 (61)	
	College graduate	804 (52)	847 (48)	
	Postgraduate	1580 (62)	1033 (38)	
**Insurance, n (%)**				<.001
	No	36 (20)	139 (80)	
	Yes	2662 (53)	2270 (47)	
	Missing	9 (24)	27 (76)	
**Income, n (%)**				<.001
	<US $20,000	209 (33)	378 (67)	
	US $20,000-US $35,000	230 (45)	303 (55)	
	US $35,000-US $50,000	291 (43)	324 (57)	
	US $50,000-US $75,000	482 (52)	424 (48)	
	≥US $75,000	1318 (59)	848 (41)	
**Diabetes, n (%)**				.02
	No	2021 (50)	1895 (50)	
	Yes	658 (57)	519 (43)	
	Missing	28 (62)	22 (38)	
**High BP** ^**d**^ **, n (%)**				.03
	No	1316 (49)	1249 (51)	
	Yes	1361 (55)	1163 (45)	
	Missing	30 (45)	24 (55)	
**Heart condition, n (%)**				.06
	No	2416 (51)	2203 (49)	
	Yes	265 (57)	216 (43)	
	Missing	26 (71)	17 (29)	
**Lung disease, n (%)**				.32
	No	2312 (51)	2127 (49)	
	Yes	373 (53)	293 (47)	
	Missing	22 (69)	16 (32)	
**Depression, n (%)**				.18
	No	2009 (51)	1896 (49)	
	Yes	669 (55)	520 (45)	
	Missing	29 (60)	20 (40)	
**History of cancer, n (%)**				.21
	No	321 (51)	320 (49)	
	Yes	1413 (54)	1153 (46)	
	Missing	67 (42)	97 (58)	

a Weighted percentages based on pooled sample of 5143 adult participants, derived using weights. Significant differences in CRC screening were evaluated with Rao-Scott tests for weighted data.

b NH: non-Hispanic.

c Includes non-Hispanic (NH) American Indian or Alaska Native, NH Native Hawaiian or other Pacific Islander, and NH Multiple Races Mentioned.

d BP: blood pressure.

### Multivariable Logistic Regression Analysis

Unadjusted and adjusted odds ratios were obtained using different logistic regression analysis models (Table 4). After adjusting for sociodemographic characteristics, access to OMR, and medical conditions (high blood pressure and diabetes), non-Hispanic Black adults reported significantly higher odds of CRC screening compared with non-Hispanic White adults (OR 1.76, 95% CI 1.22‐2.53).

**Table 4.** Unadjusted and adjusted odds ratios of colorectal cancer screening between race/ethnicity groups.

		Odds ratios	Lower limit (95% CI)	Upper limit (95% CI)	*P* value
**Model 1: Unadjusted** ^a^					
	NH White (reference)	—^b^	—	—	—
	Hispanic	0.46	0.33	0.63	<.001
	NH Black or African American	1.08	0.79	1.46	.63
	NH Asian	0.58	0.33	1.02	.06
	NH other	0.57	0.30	1.09	.09
**Model 2: Adjusted**^c^ **for access online medical records**					
	NH White (reference)	—	—	—	—
	Hispanic	0.49	0.37	0.66	<.001
	NH Black or African American	1.14	0.84	1.56	.40
	NH Asian	0.58	0.33	1.02	.06
	NH other	0.58	0.30	1.10	.10
**Model 3: Adjusted**^d^ **for gender and sociodemographic variables (age, education, and insurance)**					
	NH White (reference)	—	—	—	—
	Hispanic	0.83	0.58	1.19	.31
	NH Black or African American	1.65	1.16	2.35	.01
	NH Asian	0.76	0.38	1.51	.43
	NH other	0.99	0.50	1.93	.97
**Model 4: Adjusted**^e^ **for gender, sociodemographic variables, and access online medical records**					
	NH White (reference)	—	—	—	—
	Hispanic	0.90	0.64	1.26	.54
	NH Black or African American	1.74	1.22	2.48	.002
	NH Asian	0.78	0.40	1.52	.46
	NH other	0.99	0.52	1.91	.98
**Model 5: Adjusted**^f^ **for gender, sociodemographic variables, access online medical records, and medical conditions (high blood pressure and diabetes)**					
	NH White (reference)	—	—	—	—
	Hispanic	0.89	0.63	1.25	.49
	NH Black or African American	1.76	1.22	2.53	.003
	NH Asian	0.78	0.40	1.53	.46
	NH other	0.93	0.49	1.77	.82

a Unadjusted odds ratio (OR). Model 1 included only the main predictor (race and ethnicity [ie, non-Hispanic (NH) or Hispanic]).

b Not applicable.

c Adjusted OR. Model 2 adjusted for access online medical records.

d Adjusted OR. Model 3 adjusted for age, gender, education, and insurance.

e Adjusted OR. Model 4 adjusted for access online medical records, age, gender, education, and insurance.

f Adjusted OR. Model 5 adjusted for access online medical records, age, gender, education, insurance, and medical conditions (high blood pressure and diabetes).

Access to OMRs was associated with higher odds of CRC screening, with individuals who accessed their OMR at least once having 1.89 times the odds of CRC screening compared with those who never used an OMR (95% CI 1.45‐2.46). Increasing age was also associated with higher odds of CRC screening, with 1.18 times the odds for each additional year of age (95% CI 1.16‐1.21). In addition, individuals with postgraduate degrees had significantly higher odds of CRC screening (OR 2.65, 95% CI 1.28‐5.47) than those with a high school degree or less. Having insurance was strongly associated with higher odds of CRC screening, with individuals having 3.69 times greater odds of CRC screening if they had insurance compared with those with no insurance (95% CI 2.34‐5.82).

## Discussion

### Summary of Findings

Our study was designed to examine, via cross-sectional survey, the association between accessing an OMR and CRC screening behavior among age-eligible adults in the US general population, with specific attention to disparities according to race and ethnicity. Early detection through routine CRC screening has the potential to prevent more than 50% of CRCs, reduce advanced stage diagnoses, and increase the effectiveness of treatment for at least average-risk adults in the United States [39]. Racial and ethnic minorities presently experience elevated rates of CRC incidence and mortality, underscoring the importance of intensified promotion efforts for CRC screening within these communities [40]. Our study results revealed associations between CRC screening behavior and current OMR access, suggesting that OMR utility may potentially contribute to utilization of CRC screening among age-eligible adults. Findings also revealed nearly twice the odds of CRC screening utilization among non-Hispanic Black adults when compared with non-Hispanic White adults after adjusting for several factors. Further research is needed to explore these associations.

Our study corroborates report on mitigation in the gap between CRC screening rates of US adults self-identifying as non-Hispanic Black and others [41]. Non-Hispanic Black individuals in our study reported even higher odds of CRC screening when compared with non-Hispanic White individuals, after adjusting for various sociocontextual and medical factors. While significant, these findings do not convey rates of up-to-date CRC screening according to USPSTF guidelines, nor align with CRC screening rates reported by safety net clinics serving adults from lower-resourced communities. For example, abnormal results of stool-based testing (ie, FITs, etc) require follow-up examination via visual inspection colonoscopy [7]. With remaining disparities in CRC-specific morbidity and mortality among racial and ethnic minorities, efforts to eliminate CRC screening disparities should continue to address issues across the care continuum from screening uptake, quality, and follow-up of abnormal screening results [42]. Findings do suggest that health equity–centered strategies should be continued for non-Hispanic Black adults and replicated for men, adults aged 45‐49 years, other racial and ethnic minority groups, the uninsured, and other groups experiencing poorer CRC screening–related outcomes and continued disparities in CRC incidence and mortality [43,44].

Lower CRC screening rates based on other sociodemographic characteristics (ie, age, lower educational attainment, and being uninsured) and not having a preexisting medical condition suggest the need for personalized, patient-centered approaches [45]. The use of technology may still be an underutilized tool with potential to increase CRC screening rates (29%) among younger adults (ie, adults pre-Medicare, newly CRC screening age-eligible adults aged 45‐49 years, millennials who are nearly CRC screening age-eligible, etc) and adults requiring lower literacy and lower-cost CRC screening options (ie, adults with lower educational attainment, adults who are uninsured, etc). Web-based dissemination of CRC educational materials has already proven successful when delivering preparatory instructions (eg, on how to complete at-home stool-based testing or colonoscopy preparation) or reminding patients to complete CRC screening [23,24,46,47]. Exploring other web-based interventions for individuals who are age-eligible for CRC screening could reduce lower screening rates among diverse subgroups and provide helpful insights into the development and design of more effective health communication strategies.

Results from our study support readily available resources, such as OMRs, as potentially effective tools to promote CRC screening. Despite known disparities, OMRs are reportedly used by 90% of US health care systems and constantly increasing patient enrollment [48-50]. With user instruction, the patient portal may be an ideal tool for increasing patient-provider communication regarding CRC screening completion [51]. Notably, while our study does identify stronger predictors of CRC screening behaviors, such as insurance status and educational attainment, patient portal use is much more easily accessible and operationalized among a patient population than, for instance, expanding insurance access or increasing patients’ educational attainment. Promoting age-eligible CRC screening information and locale of free or reduced cost screening programs or events via the patient portal may potentially circumvent disparities based on insurance status, particularly within federally qualified health care centers that provide services despite a patient’s ability to pay or on a sliding-fee scale. Disseminating information on the various CRC screening modalities (ie, at-home stool-based tests, colonoscopy, etc) may also be helpful to address patient fear or concerns for procedural discomfort. Furthermore, review of the literature provides evidence that tailored or targeted interventions including patient education and access to screening are most effective for increasing CRC screening [52,53]. More research exploring OMR utility with socially vulnerable populations is needed.

Given its substantiation in previous literature, plus its clear potential to bridge sociodemographic divides that exist among the adult population for CRC, the OMR is an ideal tool for dissemination of tailored, language-concordant material promoting awareness of CRC and CRC screening completion. However, researchers have yet to identify suitable, OMR-based interventions for age-appropriate CRC screening promotion across health care settings [54-58]. By leveraging technology and facilitating access to OMRs, health care providers can potentially improve communication with patients and encourage CRC screening completion.

### Limitations

It is essential to acknowledge that our study findings are based on the analysis of the HINTS dataset and subject to limitations inherent in cross-sectional, survey-based research, including the nature of the data to restrict the ability to establish causality. Notably, we were unable to accurately determine risk for CRC based on limited survey items assessing genetic predisposition or family history of CRC. Self-report of access to OMRs and CRC screening may also have limited our results, as well as the inability to infer education on CRC alongside reported OMR use. In addition, dichotomization of CRC screening into ever or never categories might imply that patients who are not up-to-date with screenings per USPSTF recommendations have similar screening behaviors as those who are in concordance with guidelines. Also important to note, lower rates of CRC screening among younger individuals in this sample may be partially explained by the years of HINTS survey data analyzed (2018‐2020) not coinciding with the 2021 update by the USPSTF to expand the recommended age of CRC screening to include average-risk adults aged 45‐49 years [59]. Further research is needed to explore underlying mechanisms and to develop targeted interventions to reduce disparities in CRC screening based on risk status and promote the use of OMRs to enhance preventive care and early detection of CRC.

### Strengths

Access to the web and use of technology have now been identified as social determinants of health [32,60,61]. Our study is among the first to present findings on the utility of OMRs for CRC screening among the general population. Our study provides behavioral and sociocontextual information related to addressing this social determinant of health and hopefully reducing the second leading cause of cancer-related deaths through early detection of CRC. The generalizability of our study results is strengthened by the use of a nationally representative sample from HINTS, which includes diverse sociodemographic groups across the United States. However, the findings may be particularly relevant to populations already engaged with digital health tools, such as OMRs. Future research should explore interventions that expand OMR access to underrepresented and younger populations to ensure broader applicability of these results.

### Suggestions for Further Research

Utilization of web-based technology to promote CRC screening presents an ideal opportunity for health care centers to expand on existing behaviors, including web use for cancer health information seeking and cell phone use [62]. Future studies should explore and design language-concordant, patient-centered CRC prevention interventions through web-based patient portals for priming and promoting CRC screening completion among age-eligible adults [63-65]. These studies should also examine barriers at the systemic level (eg, readiness to implement cancer prevention interventions for screening age-eligible patients, patient navigation, etc), provider level (eg, communication strategies for motivating screening adherence, staff capacity, etc), and patient level (addressing facilitators and barriers to adhere to current recommendations as well as preference for web-based information delivery) [54-56,66,67]. Finally, given the recent USPSTF recommendations to expand CRC screenings to the 45‐49 years age group, targeted approaches to how this age group might benefit and interact with web-based patient portals will be especially relevant to future research in CRC screening promotion.

### Conclusions

OMRs are underutilized resources that may potentially accelerate cancer education, awareness, and screening utilization. More than ever, there exists an ideal opportunity to expand culturally inclusive client communication to promote age-appropriate CRC screenings beyond conventional, print-based materials typically offered from health care centers [22,54-58]. This study provides findings of client-centered, behavioral (access to OMR), and sociocontextual information (age, gender, socioeconomic status, and preexisting medical conditions) directly related to addressing social determinants of health and potentially reducing the second leading cause of cancer-related deaths in the United States through early detection. These findings are critical for designing and implementing future interventions that can reduce existing CRC screening–related disparities and more effectively leverage existing health care resources.

## Supplementary material

10.2196/53229 Checklist 1 STROBE (Strengthening the Reporting of Observational Studies in Epidemiology) checklist.

## Abbreviations

CRC: colorectal cancer
FIT: fecal immunochemical test
gFOBT: guaiac-based fecal occult blood test
HINTS: Health Information National Trends Survey
IRB: institutional review board
OMR: online medical record
OR: odds ratio
US: United States
USPSTF: United States Preventive Services Task Force
